# Use of *In Vitro* Assays to Assess Immunogenicity Risk of Antibody-Based Biotherapeutics

**DOI:** 10.1371/journal.pone.0159328

**Published:** 2016-08-05

**Authors:** Marisa K. Joubert, Meghana Deshpande, Jane Yang, Helen Reynolds, Christine Bryson, Mark Fogg, Matthew P. Baker, Jonathan Herskovitz, Theresa J. Goletz, Lei Zhou, Michael Moxness, Gregory C. Flynn, Linda O. Narhi, Vibha Jawa

**Affiliations:** 1 Department of Attribute Sciences, Amgen Inc., Thousand Oaks, California, United States of America; 2 Department of Clinical Immunology, Amgen Inc., Thousand Oaks, California, United States of America; 3 Antitope Limited, Babraham Research Campus, Cambridge, United Kingdom; 4 Department of Clinical Immunology, Amgen Inc., Seattle, Washington, United States of America; 5 Department of Medical Sciences, Amgen Inc., Thousand Oaks, California, United States of America; University of California, San Francisco, UNITED STATES

## Abstract

An *In Vitro* Comparative Immunogenicity Assessment (IVCIA) assay was evaluated as a tool for predicting the potential relative immunogenicity of biotherapeutic attributes. Peripheral blood mononuclear cells from up to 50 healthy naïve human donors were monitored up to 8 days for T-cell proliferation, the number of IL-2 or IFN-γ secreting cells, and the concentration of a panel of secreted cytokines. The response in the assay to 10 monoclonal antibodies was found to be in agreement with the clinical immunogenicity, suggesting that the assay might be applied to immunogenicity risk assessment of antibody biotherapeutic attributes. However, the response in the assay is a measure of T-cell functional activity and the alignment with clinical immunogenicity depends on several other factors. The assay was sensitive to sequence variants and could differentiate single point mutations of the same biotherapeutic. Nine mAbs that were highly aggregated by stirring induced a higher response in the assay than the original mAbs before stirring stress, in a manner that did not match the relative T-cell response of the original mAbs. In contrast, mAbs that were glycated by different sugars (galactose, glucose, and mannose) showed little to no increase in response in the assay above the response to the original mAbs before glycation treatment. The assay was also used successfully to assess similarity between multiple lots of the same mAb, both from the same manufacturer and from different manufacturers (biosimilars). A strategy for using the IVCIA assay for immunogenicity risk assessment during the entire lifespan development of biopharmaceuticals is proposed.

## Introduction

Immunogenicity to protein based biotherapeutics is a complex process that involves numerous patient and product specific factors [1,2]. Monoclonal antibodies (mAbs) are a major class of protein biotherapeutics that have many product specific factors that are critical for the quality of the drug product. These critical quality attributes may include: variations in the primary sequence, host-cell specific post-translational modifications, the presence of host cell proteins, formulation changes, aggregation, chemical modifications (oxidation, deamidation, or glycation), and changes in protein structure. Some critical quality attributes of mAb drug products have been suggested to affect patient safety through enhancing the sequence based risk of immunogenicity, although the exact contribution of specific types of attributes is not known.

T-cell dependent responses are the primary drivers of the long-term affinity matured immune response to biologics in the clinic [3,4]. Several formats of cell-based assay platforms have been explored to assess the risk of immunogenicity. These include assay systems using: whole blood, peripheral blood mononuclear cells (PBMC), CD8^+^-depleted PBMC, immortalized cell lines, dendritic cells/monocytes/macrophages co-cultured with autologous CD4^+^ T-cells, and artificial lymph node systems, to name a few [5–12]. These assays aim to replicate the response of typical cells associated with propagation of an immune response via monocytes, dendritic cells, T- and B-cells. Various biological outcomes can be measured at different stages of immune cell activation in these *in vitro* assays including: cytokine secretion, expression of cell surface markers of activation, identification of HLA-DR bound peptides, signal transduction events, phagocytosis by antigen presenting cells (APC), and proliferation.

Historically, *in vitro* immune cell-based assays have been used for testing rejection during transplantation [13]. These assays have also been used during pre-clinical development of immune modulatory therapeutics to understand human responses using the appropriate assay with immune cells as components [12,14,15]. More recently, PBMC-based assays have been used to describe the potential immunogenicity of protein molecules and unwanted product quality attributes for both early and late phase responses [8,16–21]. In one study, highly aggregated antibodies used therapeutically were shown to enhance the cytokine secretion and T-cell proliferation responses of a population of PBMC from healthy human donors [8].

Many different critical quality attributes are assessed as part of the product profile evaluation during early development. These attributes and changes in formulations that warrant risk assessments could be evaluated by adapting and applying *in vitro* human PBMC assays [22]. A kinetic analysis of responses following stimulation with the protein antigens of interest could be useful for detecting both low and high frequency antigen-specific effector cells. T-cell responses after stimulation *in vitro* can be assessed by enzyme-linked immunosorbent assay (ELISA), multiplex cytokine analysis, enzyme-linked immunospot spot (Elispot), proliferation, and flow cytometry analysis of cellular activation markers. With this strategy, the risk of immunogenicity could potentially be predicted much earlier than the clinical outcome [5–8,23].

In this work, we assessed one type of cell-based IVCIA assay as a potentially valuable method for predictive immunogenicity risk assessment. We evaluated the response in the assay to mAbs with different sequences and known observed clinical immunogenicity rates, examined the response to several mAb attributes, and compared the response to different drug product lots. Multiple readouts from this individual assay were evaluated to determine the best readout or combination of readouts to obtain the most effective relative risk ranking. Overall, these results indicate that this type of IVCIA assay might be readily employed for relative immunogenicity risk assessment of sequence and other manufacturing associated attributes during the drug product development lifecycle.

## Materials and Methods

### Materials

Human IgG_1_ monoclonal antibodies (Herceptin, Campath, Xolair, Erbitux, Avastin, Rituxan, Remicade, and Humira) are currently (or were previously) commercially available as highly purified solutions used therapeutically. Purified human IgG_2_ monoclonal antibodies (mAb1 and mAb2), IgG_1_ monoclonal antibodies (mAb3, ABP 501, and ABP 980), a fusion protein of an enzyme linked to the Fc domain of a monoclonal antibody (FP1), three mutants of FP1 (mutant-1 (XXLL**D**VLXX), mutant-2 (XXL**W**NVLXX) and mutant-3 (XXL**FD**VLXX)), and the fusion protein alone (FP1 (no Fc)) were supplied by Amgen as high concentration solutions. The form of the protein before stress treatment is called “original” throughout the manuscript.

### Modified Sample Preparation

#### Generation of Aggregates

Stirring induced aggregates were generated to resemble those that are created during the manufacturing, shipping and administration of biotherapeutics, as described previously [8]. Herceptin, Campath, Xolair, Erbitux, Avastin, Rituxan, mAb1, Remicade, mAb2, and Humira were diluted to 1 mg/mL in 10 mM Acetate pH 5.0 (A5 buffer). The protein sample (2 mL) was then stirred with a 6 X 6 mm Teflon stirrer bar at ~ 700 rpm, creating a vortex, in a 5 mL glass vial capped and placed vertically on a magnetic stirrer plate for 20 h (labeled as stir-20h). The purity of the samples was assessed by denaturing SDS-PAGE and silver staining (SilverXpress, Invitrogen, Paisley, UK), and indicated no significant degradation or detectable contaminating species (data not shown).

#### Generation of Glycated mAbs

Avastin, mAb1, and Humira were diluted to 2.5 mg/mL with a volume of phosphate buffer saline stock sugar solution, galactose, glucose, or mannose (0.5–1.6 M), to reach a targeted forced glycation level of 40% (0.4 mole sugar/mole mAb) over the initial content for each mAb before treatment, as previously described [24]. The mAbs were also diluted and incubated similarly with 1.0 M sorbitol to prepare a control sample that was treated with sugar, but without an increase in the level of glycation. Samples were incubated at 37°C for 8 hr (Avastin and Humira) or 21 hr (mAb1). After incubation, the forced glycation samples were dialyzed into A5 buffer. The protein concentration of each sample was determined by absorbance at 280 nm. The integrity of the samples was verified by size exclusion chromatography and the level of forced glycation was determined by reverse phased liquid chromatography/mass spectrometry (data not shown), as previously described [24].

The samples before aggregation or glycation treatment were used to monitor the response to the original form of the mAb (labeled as original in the text and Figs).

### Endotoxin Testing

All protein samples and buffers were assessed for endotoxin levels by a LAL (Limulus amebocyte lysate) test with the Charles River Endosafe-PTS (Charles River, Wilmington, MA) system prior to being used in the biological assay. The analysis was performed according to the manufacturer’s instructions. All samples were found to have endotoxin levels that were within the limit acceptable for these cell based assays (< 1.00 EU/mL).

### *In Vitro* Comparative Immunogenicity Assessment (IVCIA) Assays

An IVCIA assay was performed to assess CD4^+^ T-cell responses by Antitope (Antitope Ltd., Babraham Institute, Cambridge, UK) to monitor the response in a 50 donor population, as described previously [5,6,8]. Briefly, PBMC were isolated from blood drawn within 24 h from healthy donors from the UK National Blood Transfusion Service (Addenbrooke’s Hospital, Cambridge, UK) according to approval granted by Addenbrooke’s Hospital Local Research Ethics Committee, using Lymphoprep (Axis-shield, Dundee, UK) density centrifugation. CD8^+^ T-cells were depleted using CD8^+^ RosetteSep (StemCell Technologies Inc, London, UK). Donors were characterized by identifying HLA-DR haplotypes using an HLA SSP-PCR based tissue typing kit (Biotest, Solihull, UK) or the HISTO Spot SSO HLA typing method. PBMC were frozen and stored in liquid nitrogen until required. A population of 50 human donors was selected to best represent the number and frequency of HLA-DR allotypes expressed in the world population and to include all major HLA-DR allotypes (individual allotypes with a frequency >5% expressed in the world population) (S1 Fig). Several experiments were performed at different times, so not all mAbs were tested in the same set of 50 donors. PBMC were resuspended in AIM-V medium (Life Technologies, Carlsbad, CA) at a concentration of 4–6 X 10^6^ cells/mL. PBMC were added to the 24-well proliferation plates (2 mL final volume) and 48-well cytokine secretion plates (1 mL final volume) at a final concentration of 2–4 X 10^6^ cells/mL, and then challenged with a final concentration of 40 μg/mL of each sample. This concentration was optimized during previous experiments based on what the cells can tolerate and their response to a protein with associated attributes, and was not intended to simulate dosing conditions in the clinic. For each donor, responses to a negative control, consisting of medium-treated cells (referred to as the background response), and several reproducibility controls, including keyhole limpet hemocyanin (KLH, Pierce, Cramlington, UK), phytohemagglutinin (PHA, Sigma, Poole, UK), and a benchmark clinical control (the humanized A33 antibody produced at Antitope), were also included. T-cell proliferative responses were monitored on days 5, 6, 7, and 8 for all experiments except the biosimilars comparison (day 7 only). The viability of PBMC from 10 representative donors for each sample was assessed by trypan blue dye exclusion on day 7 after challenge, and found to be greater than 90% for all samples.

To evaluate the ability of an IVCIA assay to potentially be applied in a manufacturing setting in a smaller group of donors (n≤12), a slightly modified IVCIA assay was also performed at Amgen in a similar fashion (with a few modifications that are described below). This format was used for the glycation experiment, lot-to-lot comparison, and biosimilars comparison. Whole blood from healthy naïve human donors was supplied by Amgen’s Environmental Health and Safety department (EH&S) according to local ethical practices. Written consent was obtained from each donor. PBMC from up to 12 donors per experiment (see individual experiment for exact number) were isolated, cryopreserved in freezing medium, thawed and plated on the day of the study, and stimulated as previously described [8]. In brief, PBMC were plated at 2.5 X 10^6^ cells/mL in a total volume of 200 μL of RPMI growth media at 37°C containing: 89% RPMI Medium 1640, 10% heat-inactivated fetal bovine serum, and 1% penicillin/streptomycin/L-glutamine (Life Technologies, Carlsbad, CA) in 96-well culture plates. An overnight acclimatization step was performed for the glycation experiment only so that PBMC and adherent monocytes could be challenged together. Cells were then challenged with a final concentration of 40 μg/ml (glycation and lot-to-lot comparison) or 100 μg/mL (biosimilars comparison) of the different mAb samples. The sorbitol control was added at an equivalent volume to the glycated mAb samples. The glycated and sorbitol samples were centrifuged at 13,000 rpm for 2 min before challenge. All donors had greater than 81% viability before plating (except 2 donors that were 71–77%). Plates were placed in a 5% CO_2_ incubator at 37°C for 20 h (early phase) or 7 days (late phase). A negative control, consisting of medium-treated cells, and positive controls, including lipopolysaccharide (LPS) and PHA, were also tested.

### Adherent Monocyte Evaluation

For the glycation experiment only, a duplicate plate of PBMC from the same donors was isolated, plated and acclimatized as described above. After the overnight acclimatization step, non-adherent cells were removed from the culture by removing all standing liquid. Adherent monocytes in the bottom of each well were washed two times with 200 μl of RPMI growth media. 200 μl of fresh RPMI growth media was then added to each well. Adherent monocytes were challenged with the original and glycated mAbs at 40 μg/ml, as described in the PBMC simulation section.

All cell derived supernatants were frozen at appropriate time points and stored at -70°C for multiplex cytokine analysis.

### T-cell Proliferation

Culture plates for T-cell proliferation assays were incubated in a 5% CO_2_ incubator at 37°C for up to 8 days. On days 5, 6, 7 and 8, the cells in each well of the plate were gently resuspended and transferred to a round bottom 96-well plate. The cultures were then pulsed with 0.75 μCi [^3^H]-thymidine (Perkin Elmer, Beaconsfield, UK) in AIM-V culture media, and incubated for 18 h. Cultures were harvested onto filter mats (Perkin Elmer, Beaconsfield, UK) using a TomTec Mach III cell harvester (Hamden). Counts per minute (cpm) for each well were determined by scintillation counting using a 1450 Microbeta Wallac Trilux Liquid Scintillation Counter (Perkin Elmer, Beaconsfield, UK) in paralux, low background counting. For data analysis, the stimulation index (SI) was calculated by dividing the proliferative response of the test well (cpm) by the proliferative response of the medium-only treated well (cpm) for each donor. A response was considered positive if the SI was greater than 2.0 (SI≥2.0) and statistically significant (*p*<0.05) as compared to medium-only treated wells using an unpaired two sample student’s t-test. The percentage of donors that responded (% donors responding) was calculated by taking the number of donors that had a positive response (SI≥2.0, *p*<0.05) over the entire time course (5–8 days) as a percentage of the total number of donors that were tested. Positive thresholds in all assays were previously established based on statistically derived cut points. Extensive assay development and previous studies have shown that this is the minimum signal to noise threshold allowing maximum sensitivity without detecting large numbers of false positive responses or omitting subtle immunogenic events.

### IL-2 and IFN-γ Elispot

For the IL-2 Elispot readout, anti-human IL-2 Elispot plates (Millipore, Watford, UK) that were pre-wetted and coated overnight with IL-2 capture antibody (R&D Systems, Abingdon, UK) were used. PBMC were added to the IL-2 Elispot plates at a final concentration of 2–4 X 10^6^ cells/mL, and then challenged with 40 μg/mL of each sample. IL-2 Elispot Plates were incubated 8 days before developing according to the manufacturer’s instructions (R&D Systems). Dried plates were scanned on an Immunoscan Analyzer (Cellular Technology Limited, Shaker Heights, Ohio) and the number of spot forming cells (SFC) per well were determined using Immunoscan Version 4 software. The SI was calculated by dividing the number of IL-2 secreting cells in the test well (SFC) by the number of IL-2 secreting cells in the medium-only treated well (SFC) for each donor. A response was considered positive if the SI≥2.0 and used to calculate the percentage of donors that responded similar to the T-cell proliferation section.

For the IFN-γ Elispot readout, Elispot was performed using a kit according to the manufacturer’s instructions (Mabtech, Stockholm, Sweden). Briefly, PBMC from 6 donors were added at a concentration of 1.5 X 10^6^ cells/ml to a 96-well Elispot plate and then challenged with 40 μg/mL of the original or glycated samples. The IFN-γ Elispot plates were incubated for 7 days and then developed according to the manufacturer’s instructions. Negative controls testing medium only or no cells as well as a PHA positive control were also included. The dried plates were scanned on an AID Elispot Reader version 7.0 (AID GmbH, Strassberg, Germany), and SFC were determined using AID EliSpot Software v5.0. For data analysis, to highlight the potential differential response of glycated mAbs, the SI was calculated as the SFC for donors treated with glycated mAbs divided by the SFC for donors treated with the original mAbs before glycation treatment. An SI ≥ 2.0 was considered positive.

### Multiplex Cytokine Analysis

Multiplex cytokine analysis was performed on culture supernatants by Luminex technology using Milliplex human panel kits (EMD Millipore, Billerica, MA) and a Luminex FlexMAP 3D instrument with the XPonent version 4.0 software, as described by Joubert et al. [8]. Cell culture supernatants were thawed and then centrifuged at 1200 rpm for 5 min before testing. For analysis of the PBMC supernatants at the early phase (after 20h incubation), the following cytokines were monitored: IL-1α, IL-1β, IL-1ra, IL-6, IL-8, IL-10, MCP-1, MIP-1α, MIP-1β, TNF-α, and TNF-β. For analysis of the PBMC supernatants at the late phase (after 7 days of incubation), the following cytokines were monitored: IFN-γ, IL-2, IL-4, IL-5, IL-6, IL-7, IL-8, IL-10, IL-12p40, IL-12p70, IL-13, and TNF-α. For data analysis, the SI was calculated by dividing the amount of cytokine detected (pg/mL) in the treated sample by the relevant control sample. The relevant control sample for the cytokine analysis was either the medium-only treated wells or the original mAb (see Fig legends for the type of baseline used for each experiment). A response was considered positive if the SI was greater than 2.0 at the early phase in PBMC (SI≥2.0), greater than 3.0 at the early phase in monocytes (SI ≥3.0), or greater than 1.9 at the late phase (SI≥1.9). Assay cut points were based on statistical analysis and/or the differential response observed to the media alone in the different assay systems used. The percentage of donors that responded (% donors responding) was calculated by taking the number of donors that had a positive SI as a percentage of the total number of donors that were tested. In all cases, the negative controls such as medium-treated cells showed a minimal response and positive controls such as LPS, PHA and/or KLH showed a very high response (SI>>3.0; only select data shown). In all cases, the percentage of donors that responded to the mAb samples was much lower than that induced by the positive controls, KLH (72–86%) and PHA (98%).

### Statistical Analysis

Statistical analysis of the proliferative responses and concentration of cytokines determined by multiplex cytokine analysis was performed using a mixed effect analysis of variance model as previously described [8]. Briefly, the model assumed correlated responses from the same donor and independent responses from difference donors. When evaluating the response to the original mAb relative to background, the model included compound (mAb), condition (original and background), and the two-way interaction between compound and condition as fixed effects, calculated for the proliferative response or each cytokine. The response to each original mAb was compared with the background response, and a one-sided p value was reported. When evaluating the response to aggregated mAbs relative to the response to the original form, the model included compound (mAb), condition (stir-20h, original and background), and the two-way interaction between compound and condition as fixed effects, calculated for the proliferative response or each cytokine. *p* values less than 0.05 were considered as statistically significant.

### Aggregate Analysis

Aggregated solutions were characterized for particle count, size distribution and morphology as described in our previous report [25]. Aggregated samples made at 1 mg/mL were diluted in A5 buffer (20-100X), degassed, and then analyzed by the appropriate instrument. Particle counting and size distribution in the micron size range was performed on a HIAC/Royco Liquid Particle Counter model 9703 with an HRLD-150 sensor and the software PharmSpec (HACH Ultra Analytics, Grants Pass, OR). Particle imaging in the micron and visible size range (≥ 125 μm) was achieved by a liquid-borne particle Micro-Flow Imaging (MFI) System DPA4100 (Protein Simple, Santa Clara, CA). All final particle numbers were adjusted for the initial dilution factor.

## Results

### The IVCIA Assay Correlates with the Rate of Clinical Immunogenicity

In order to appraise the IVCIA assay as an effective *in vitro* tool for assessing the relative risk of immunogenicity in the clinic, ten IgG1 and IgG2 biotherapeutic monoclonal antibodies, with known rates of clinical immunogenicity (Table 1) [26,27], were tested in the IVCIA assay for their ability to elicit an immune response in PBMC. An additional mAb (mAb1), which has not been tested in the clinic, was also tested and is predicted to have a rate of clinical immunogenicity that is intermediate among the selected mAbs based on *in silico* methods. It is important to note that the immunogenicity rates shown are based on the anti-drug antibody (ADA) responses associated with diverse disease indications and assay testing platforms with variable sensitivity. The responses of PBMC from 50 healthy naïve human donors were monitored 5 to 8 days for T-cell proliferation, the number of IL-2 secreting cells (Elispot), and the concentration of secreted cytokines (multiplex cytokine secretion).

**Table 1:** Biotherapeutic mAb rates of clinical immunogenicity.

*mAb*	*Generic*	*Subtype*	*Rate of Clinical Immunogenicity* ^*a*^
**Herceptin**	**trastuzumab**	**IgG1**	0.1% * ^***b***^
**Campath**	**alemtuzumab**	**IgG1**	1.9% **
**Xolair**	**omalizumab**	**IgG1**	**0.1%**
**Erbitux**	**cetuximab**	**IgG1**	5% *
**Avastin**	**bevaciszumab**	**IgG1**	0–8% *^,^ ^***c***^
**Rituxan**	**rituximab**	**IgG1**	1–23% *^,^^*c*^^,^^*d*^
**mAb1**	**NA**	**IgG2**	**NT**
**Remicade**	**Infliximab**	**IgG1**	13–27% *^,^^*c*^^,^^*e*^
**mAb2**	**NA**	**IgG2**	**12–16%** ^***f***^
**mAb3**	**NA**	**IgG1**	**14–50%** ^***f***^
**Humira**	**adalimumab**	**IgG1**	1–87% *^,^^*c*^^,^^*e*^

^***a***^ All rates were taken from the product label except in a few cases as specified below. Rates are based on the anti-drug antibody response associated with diverse disease indications and assay testing platforms with variable sensitivity. The highest observed incidence reported for each mAb (except Rituxan, see below) was used to show association in all subsequent figures.

^*b*^ Definition of symbols: *- rate based on immunogenicity in immune compromised patients; **—rate was impacted by the immunomodulatory nature of the molecule; NA—not applicable; NT—not tested in the clinic.

^*c*^ Some mAbs have a wide range of immunogenicity rates reported. In some cases, the lower incidence of the range shown has been attributed to technical limitations of the immunogenicity assays used to support the analysis and the influence of standard of care immune suppressive medications administered to the subjects. In the case of Avastin, 0% immunogenicity was observed due to these limitations (according to the label), so the incidence for Lucentis (8%) is also shown.

^*d*^ The results of several clinical studies are reported on the label for Rituxan. The majority of patients (85%) were tested in one of these clinical studies that resulted in 11% incidence of immunogenicity. This value is used to show association in all subsequent figures.

^*e*^ The highest incidences shown for Remicade and Humira were taken from references 26 and 27, respectively.

^*f*^ The rates for mAb2 and mAb3 are based on testing in early clinical trials

**Fig 1:**
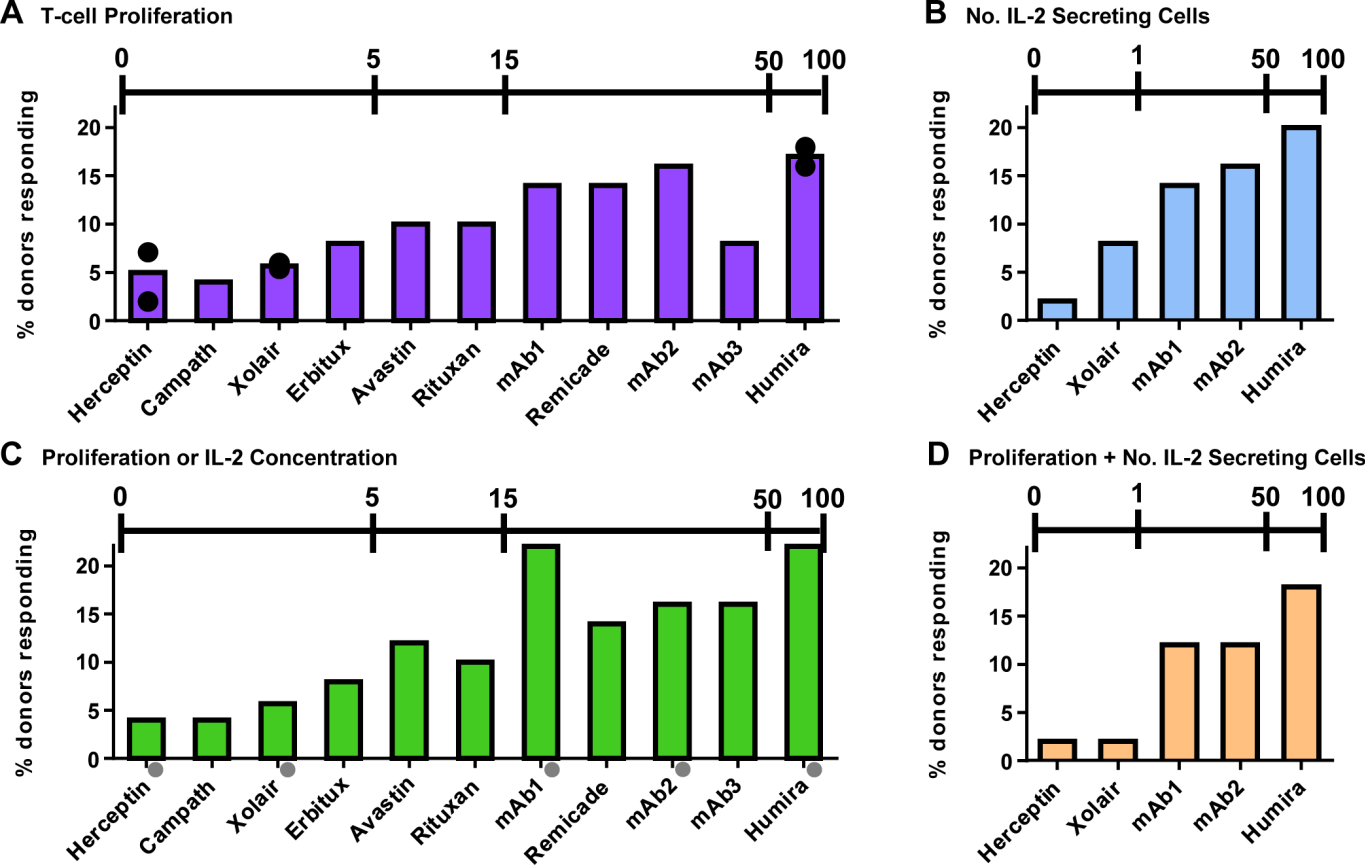
The response of CD4^+^ T-cells in the IVCIA assay agrees with the rate of clinical immunogenicity for biotherapeutic mAbs. 10 mAbs, with known rates of clinical immunogenicity, were evaluated in the IVCIA assay in a population of 50 healthy human donors over 5–8 days. mAb1 has not been tested in the clinic. Donors that responded by multiple readouts were evaluated for the most effective relative risk ranking. The percentage of donors that showed A) a positive T-cell proliferative response ([^3^H]-thymidine uptake) or B) an increase in the number (No.) of IL-2 secreting cells (Elispot) over the course of the entire study are displayed. Results were also combined to illustrate the percentage of donors that showed C) either a positive T-cell proliferation response or an increase in the concentration of IL-2 secreted (multiplex cytokine analysis) or D) a positive T-cell proliferation response and an increase in the number of IL-2 secreting cells are shown. Not all donors were tested for IL-2 for some samples (*grey circles*). A response was considered positive if the SI ≥ 2.0 (*p*<0.05) for proliferation or number of IL-2 secreting cells or the SI ≥ 1.9 for IL-2 concentration (above the background response). mAbs are ordered approximately within each graph from the lowest to the highest response in the IVCIA assay. The scale bars at the top of each graph show the highest incidence of immunogenicity reported for each mAb in Table 1. All rates are associated with diverse disease indications and assay testing platforms with variable sensitivity. *Black circles* represent duplicate experiments in different sets of 50 donors.

Fig 1 shows that the relative response of PBMC to biotherapeutic mAbs agrees with the observed rate of clinical immunogenicity for most mAbs. Individual readouts were found to correlate with the rate of immunogenicity, including the T-cell proliferative response (Fig 1A) and the number of IL-2 secreting cells (Fig 1B). The concentrations of 11 cytokines were tested on Day 7, and the cytokine IL-2, a major driver of T-cell proliferation, was found to be the most statistically significant (p<0.05) cytokine induced by the mAb samples above the background. The remaining cytokines were found not to correlate with the rate of clinical immunogenicity for most mAbs (data not shown). To evaluate if the correlation with the rate of clinical immunogenicity could be further improved, the number of donors that responded by more than one readout of the assay was also determined. In Fig 1C, donors that responded by either an increase in T-cell proliferation or IL-2 concentration were counted as responders. Including the donors that responded by IL-2 concentration improved the readout for mAb3, which has a high rate of clinical immunogenicity (up to 50%), but seemed, on first glance, to over predict the rate of immunogenicity for other molecules (mAb1) (compare Fig 1A with 1C). It is important to note that mAb1 is a unique case in that this molecule had severe aggregation and instability issues during early development and thus was not pursued as a therapeutic candidate. Therefore, the high response observed to mAb1 in Fig 1C may correctly reflect the increased risk of immunogenicity of this molecule. Similarly, donors that responded by both T-cell proliferation and an increase in the number of IL-2 secreting cells were tabulated (Fig 1D). This decreased the readout for Xolair from 8% to 2% responding donors, which agrees with the low rate of clinical immunogenicity for this molecule (0.1%) (compare Fig 1B with 1D).

To judge the reproducibility of the results, three representative mAbs (Herceptin, Xolair, and Humira), were tested in a duplicate experiment, on different days, and with different sets of 50 donors. Fig 1A (*black circles*) shows that the T-cell proliferative results were highly reproducible. For each mAb, the standard deviation between the percentages of donors that responded in each duplicate experiment was less than 4%. The correlation of the assay with the rate of clinical immunogenicity and the reproducibility of the results suggests that this assay is a reasonable approach for immunogenicity risk evaluation.

### Differences in Amino Acid Sequence can be Detected

**Fig 2:**
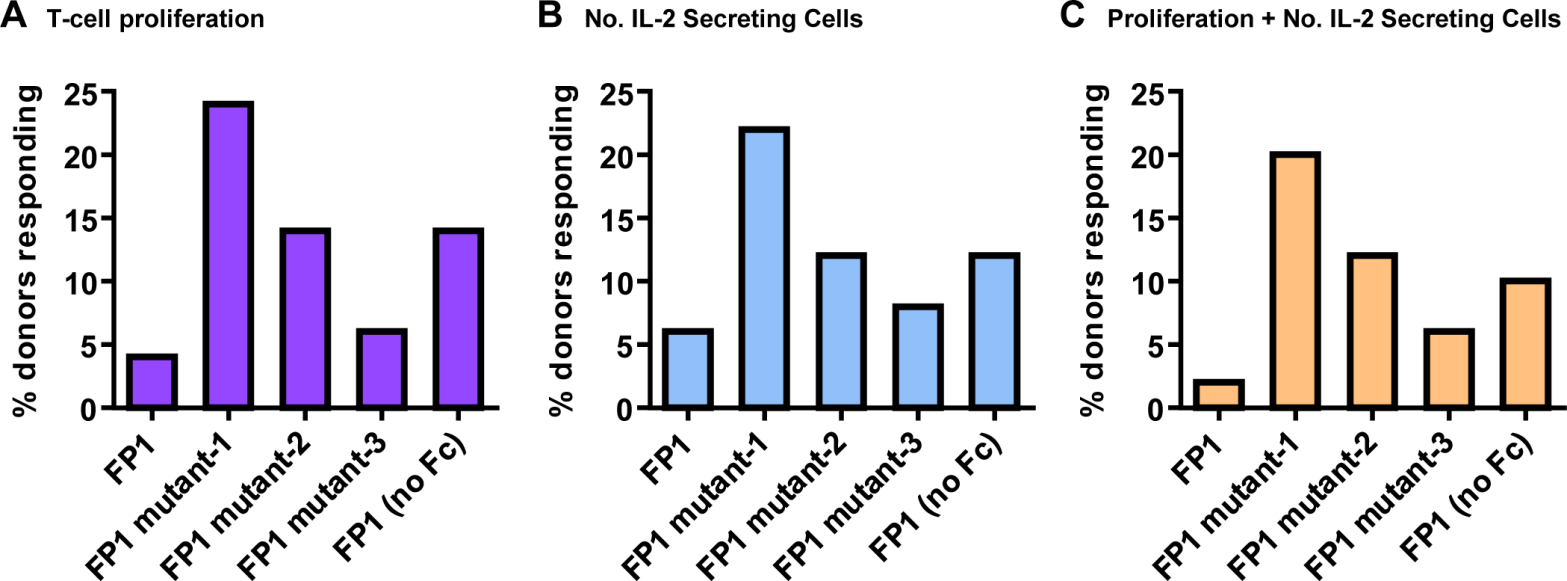
Biotherapeutics with minor differences in sequence can be distinguished by the IVCIA assay. Several sequence variants of FP1 varying by one (mutant-1 and mutant-2) or two (mutant-3) amino acids, as well as FP1 lacking the Fc domain (FP1 (no Fc)) were tested in the in the IVCIA assay using 50 healthy human donors over 5–8 days. FP1 is a biotherapeutic fusion protein of an enzyme fused to the Fc domain of a monoclonal antibody. The percentage of donors with either A) a positive T-cell proliferative response ([3H]-thymidine uptake) or B) an increase in the number (No.) of IL-2 secreting cells (Elispot), or C) both positive T-cell proliferative responses and an increase in the number of IL-2 secreting cells are shown. A response was considered positive if the SI≥2.0 (*p*<0.05) above the background response.

To determine whether the IVCIA assay can be utilized to differentiate and rank order different biotherapeutic candidates during early drug product development, the response of the assay to different sequence variants of the same biotherapeutic was tested. FP1 is a biotherapeutic fusion protein of an enzyme homodimer fused to the Fc domain of a monoclonal antibody. Several sequence variants, including proteins containing either one (mutant-1 and mutant-2) or two (mutant-3) mutations in amino acid residues, or a construct lacking the Fc domain (FP1 (no Fc)) were evaluated. All of the FP1 mutants had similar secondary and tertiary structures, and similar profiles of chemically modified amino acids. However, under accelerated stress conditions, some differences in the stability and aggregation profiles were observed (data not shown), indicating that the change in amino acid sequence may have caused differences in other attributes. FP1 has not been tested in the clinic so the rate of clinical immunogenicity for this molecule is not known. However, *in silico* immunogenicity risk assessment analysis (which evaluated the primary protein sequences for the presence of peptides with the potential to bind to human leukocyte antigen (HLA) class II alleles) showed that the non-tolerant aggretope content of FP1 and the three mutants is absent or very low (data not shown). This suggests that the risk of immunogenicity (based on the method of *in silico* analysis used) for FP1 and the three mutants is low and relatively similar. To evaluate the risk of immunogenicity of the FP1 variants in the IVCIA assay, the responses of PBMC from 50 healthy human drug naïve donors were monitored up to 8 days for T-cell proliferation, and the number of IL-2 secreting cells. Fig 2 shows that the different FP1 variants evaluated induced different levels of T-cell proliferation and number of IL-2 secreting cells in the IVCIA assay, and that the trends agreed for both readouts. The assay was found to be highly sensitive to sequence differences as little as one amino acid. For example, FP1 mutant-1 and mutant-3 differ by one amino acid yet mutant-1 gave the highest response (20% responding donors) and mutant-3 gave the lowest response (6% responding donors) of all variants. This result demonstrates the ability of the IVCIA assay to differentiate biotherapeutic proteins with minor differences in sequence during early candidate selection that might not be differentiated by *in silico* analysis alone.

### Evaluation of Aggregated Biotherapeutics in the IVCIA Assay

**Fig 3:**
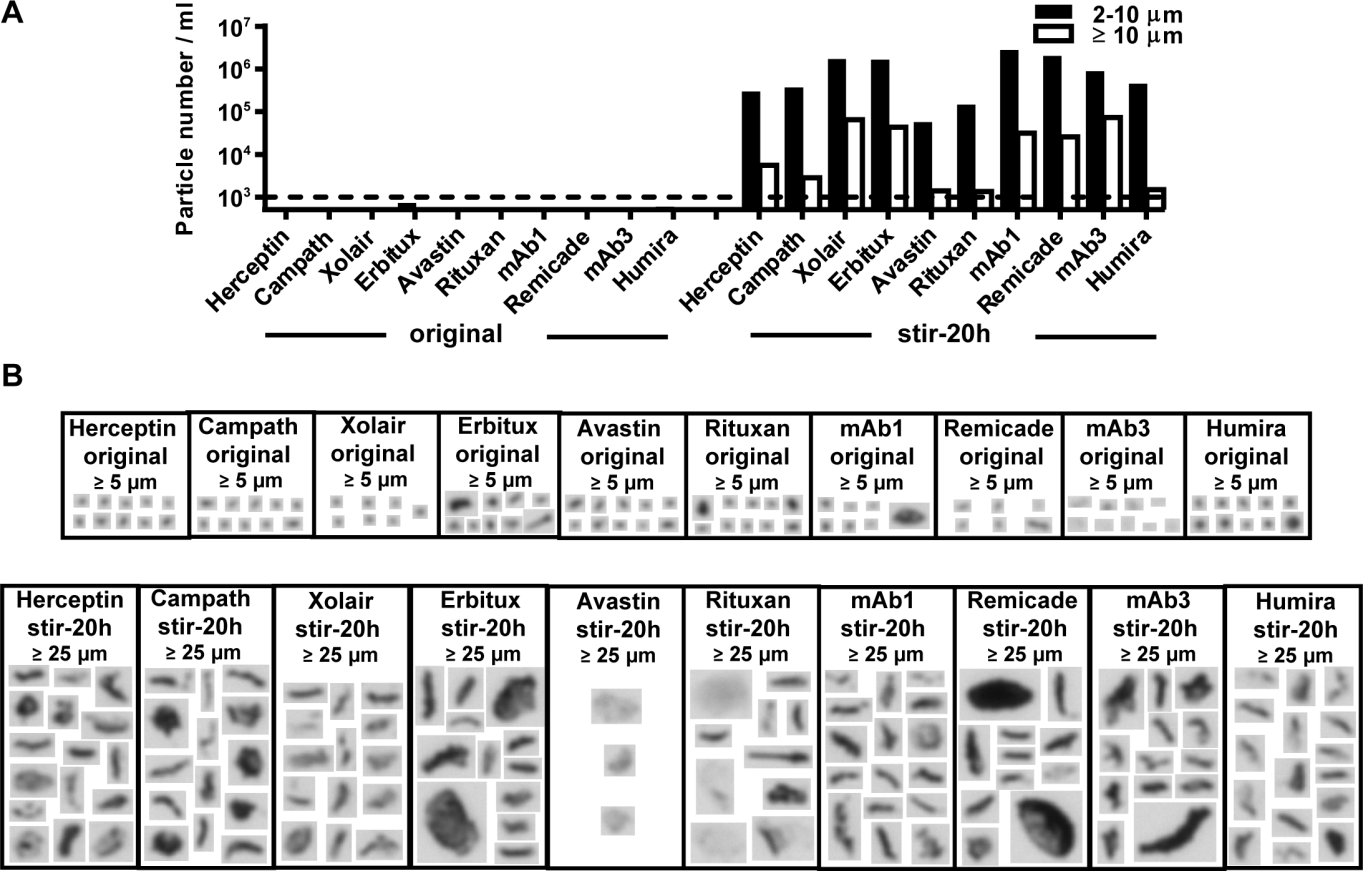
Particle counts, size distribution, and morphology of stirring induced aggregates of a broad array of antibodies. 10 mAbs (with known or predicted rates of clinical immunogenicity) were aggregated by stirring stress and then examined for their subvisible and visible aggregate content. A) Aggregates were quantitated by HIAC to determine the number and size range of particles present. Bar height represents the differential particle counts per ml in each size range (average of 3 runs). B) Aggregates images were captured on a Micro-flow Imaging System to evaluate the morphology of particles present. Representative images of the largest particles detected are shown. The size threshold indicates the lower size limit of the particles that were used for comparison. The aggregate content of the original mAbs (before stirring stress) is also shown and highlights that only a few small sized aggregates were detected.

Our group previously demonstrated that high numbers of stirring induced aggregates of mAb1 (previously referred to as mAb2 [8]) can enhance the early and late stage responses of PBMC in the IVCIA assay above the response to the original mAb1 (before stress treatment) [8,9]; in agreement with the response seen by other groups to aggregated samples *in vitro* [10,16,21,28]. To investigate if the assay can be used for evaluating aggregates across a wider range of mAbs, stirring induced aggregates of 10 mAbs were generated and characterized. Fig 3 shows the particle counts, size distribution, and morphology of the stirring induced aggregates that were generated. All protein solutions treated by stirring stress contained a high number of aggregates in the subvisible size range (Fig 3A), that were heterogeneous, amorphous, and irregular in morphology (Fig 3B). Although trends were similar between different mAbs, some differences between mAbs could be observed as well. The highest numbers of particles were seen in the Xolair, Erbitux, mAb1, and Remicade stirred samples, followed by the Herceptin, Campath, mAb2, and Humira stirred samples. Although still highly aggregated, the least number of particles, which were mostly in the 2–10 μm size range, were seen in the Avastin and Rituxan samples. As expected, the original mAb samples contained only a few small-sized aggregates. Fig 3B shows images of the largest aggregates that were detected in the original samples, which were very few in number.

**Fig 4:**
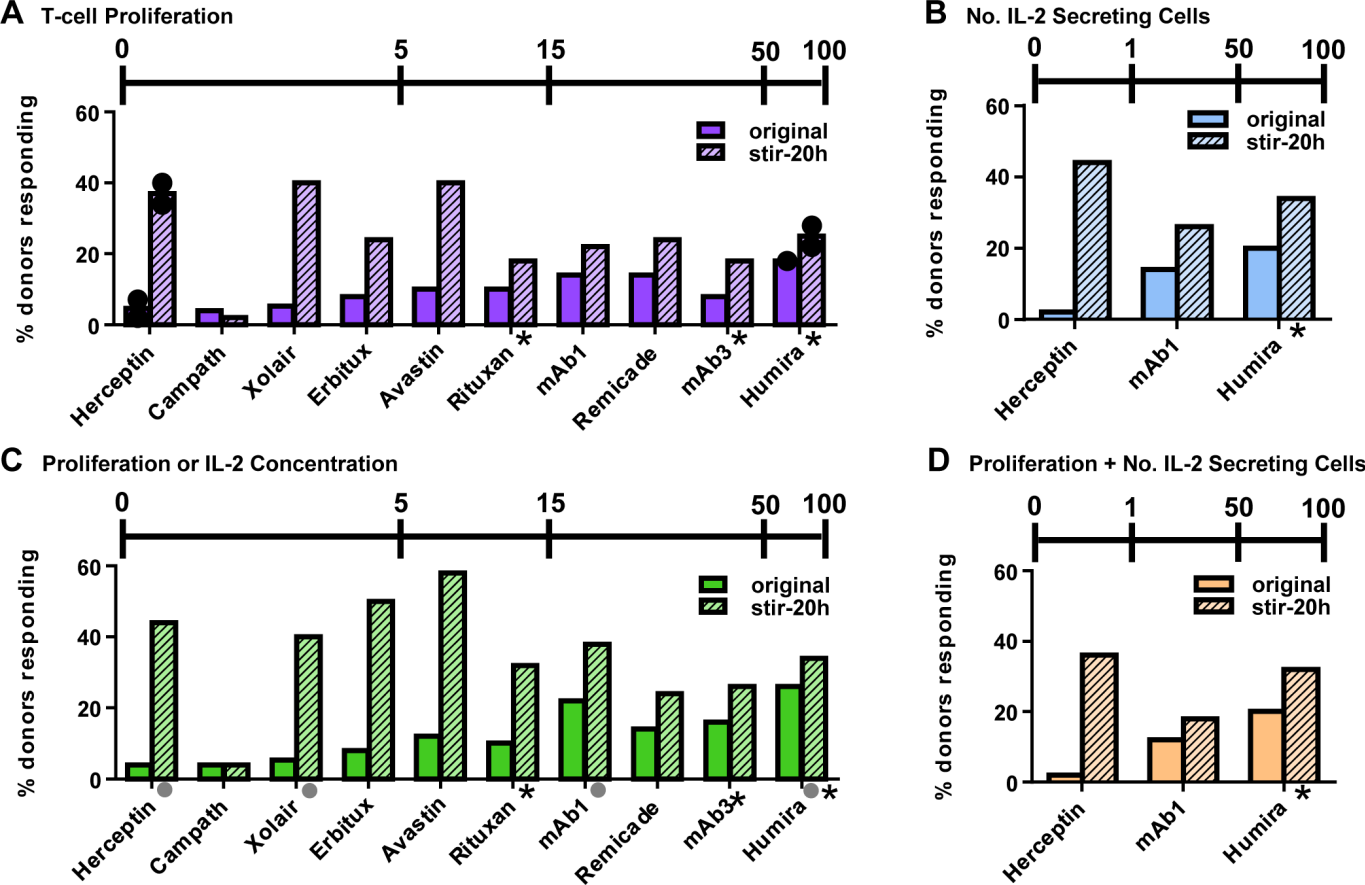
High numbers of aggregates of a wide array of antibodies can be detected by the IVCIA assay. 10 mAbs (with known or predicted rates of clinical immunogenicity) were aggregated by stirring stress and then evaluated in the IVCIA assay in a population of 50 heathy human donors. The percentage of donors that responded to the original mAb (*solid bars*) and the aggregated mAb (*striped bars*) by A) positive T-cell proliferative responses ([^3^H]-thymidine uptake) or B) an increase in the number (No.) of IL-2 secreting cells (Elispot) over the course of the entire study (5–8 days) are displayed. The percentage of donors that showed C) either a positive T-cell proliferation response or an increase in the concentration of IL-2 secreted (multiplex cytokine analysis) or D) a positive T-cell proliferation response and an increase in the number of IL-2 secreting cells are depicted. The y-axes of graphs in Figs 1 and 4 are on different scales. Not all donors were tested for IL-2 concentration for some samples (*grey circles*). A response was considered positive if the SI ≥ 2.0 (*p*<0.05) for proliferation or number of IL-2 secreting cells, or the SI ≥ 1.9 for IL-2 concentration (above the background response). Borderline responses were also included in some cases (SI≥1.9, *p*<0.05), and are shown with one asterisk. mAbs are in the same order as Fig 1. The scale bars at the top of each graph show the highest incidence of immunogenicity reported for each mAb in Table 1. All rates are associated with diverse disease indications and assay testing platforms with variable sensitivity. *Black circles* show the responses in duplicate experiments in different sets of 50 donors.

The 10 highly aggregated mAb samples were then tested in the IVCIA assay in PBMC taken from 50 healthy human donors for their ability to elicit an immune response. Fig 4 shows that stirring induced aggregates of 9 of the 10 Abs were able to enhance the response of PBMC above the original mAb for T-cell proliferation (Fig 4A), number of IL-2 secreting cells (Fig 4B), and concentration of IL-2 (Fig 4C). Aggregated Herceptin, Xolair, Erbitux, and Avastin induced the highest response in the assay, in contrast to aggregated Campath which did not induce a response. Aggregated Rituxan, mAb1, Remicade, mAb3, and Humira induced a response that was intermediate between the high and low groups. Different aggregated preparations of the same mAb tested in a different set of 50 donors yielded similar results (Fig 4A, black circles), demonstrating the reproducibility of the results.

The secretion of signature cytokines was also tested at the early and late phases and found to be enhanced in the presence of all 10 mAbs that were treated by stirring stress (Fig 4D, S2 Fig), in agreement with previous results [8]. Overall the trends in cytokine secretion were aligned with T-cell proliferation results, where stirring induced aggregates of Erbitux and Avastin induced the highest response and mAb3 induced the lowest response (above the monomer) at both the early and late phases. Some donors that showed positive T-cell proliferative responses to aggregated samples also showed enhanced secretion of the immunosuppressive cytokine, IL-10, indicating the possible involvement of regulatory T-cells or monocytes which may lead to immunological tolerance (S3 Fig). In contrast, the original mAbs induced little to no IL-10 above the background response.

### The Potential Immunological Risk from Chemical Modification

Glycation of amino acids is a potential chemical modification that can occur during the manufacture of biotherapeutics or upon administration to patients and may pose an increased risk of immunogenicity. The IVCIA assay was used to evaluate biotherapeutics that had been glycated by different types of sugars to determine the potential risk of immunogenicity of this type of modification. Three representative mAbs (Avastin, mAb1, and Humira) with low, medium and high rates of clinical (or predicted) immunogenicity, respectively, were used. mAbs were treated by galactose, glucose, mannose, and a non-glycating sugar, sorbitol, as a negative control, and then analyzed by mass spectrometry for total glycation analysis as previously described [24]. Results of the analysis showed that all samples had an increased level of glycation of approximately 40% above the original sample before glycation treatment (data not shown). This means that for the forced glycated samples, 40% of the antibodies in each sample had a single sugar attached (0.4 mole attached sugar/mole protein) above what is typically found in these specific antibody drug products, 4–10% (0.04–0.1 mole attached glucose/mole protein).

**Fig 5:**
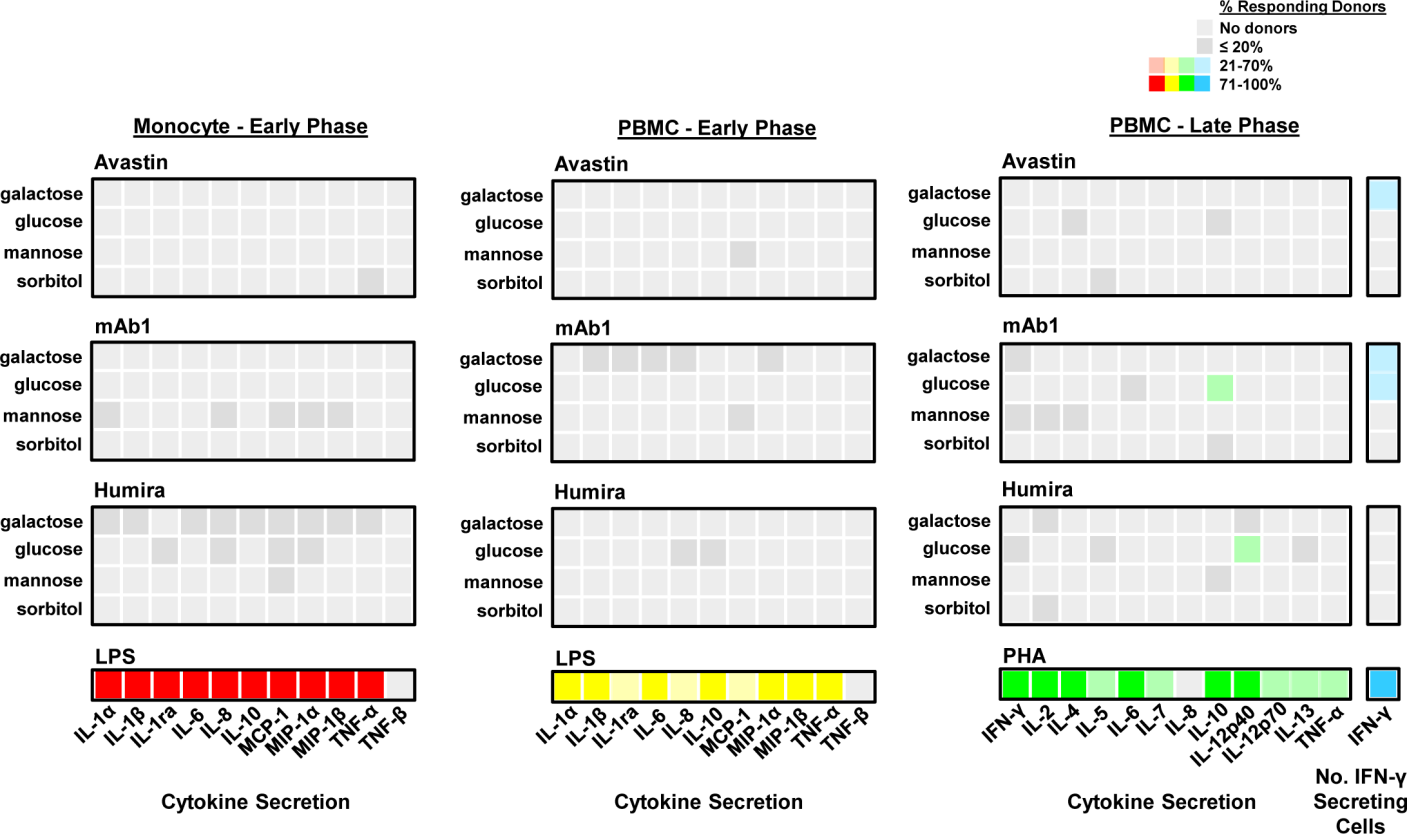
Chemical Modification by glycation does not enhance the response of PBMC in the IVCIA assay. Three representative mAbs (Avastin, mAb1, and Humira), that were treated by glycation with different sugars (galactose, glucose and mannose, and a non-glycating sugar, sorbitol), were tested in the IVCIA assay at the early (20 h) (n = 11 donors for most samples) and late (7 day) phases (n = 6 donors). Avastin, mAb1, and Humira have low, medium, and high rates of clinical or predicted immunogenicity, respectively. Heatmaps depict the percentage of donors that responded to the glycated mAbs above the original forms of each molecule (mAb before glycation treatment) and were at least two fold above the background, to highlight the differential response that might be due to glycated mAbs. The percentage of donors with increased secretion of signature cytokines in adherent monocytes at the early phase (red), and in PBMC at the early (yellow) and late (green) phases is highlighted. The percentage of donors with an increase in the number of IFN-γ secreting cells is shown on the far right (blue). The grey boxes show low level responses that were observed in less than or equal to 20% of donors, in contrast to colored boxes (red, yellow, green and blue) that show responses in a greater number of donors (40–100%).

Glycated mAbs were then evaluated in the IVCIA assay in 11 healthy human donors for their ability to stimulate an immune response in PBMC. For this experiment only, both PBMC and adherent monocytes (from the same donors) were tested to determine the potential response of a specialized cell type to glycated mAbs. Only the secretion of signature cytokines was further monitored for evaluating attribute related changes, as this readout was found to be sufficient for sensing the response to aggregates, which could be detected by cells of the innate immune system as soon as the early phase (Fig 4D and S2 Fig) [8]. Fig 5 is a heatmap representation of the percentage of donors that responded to the glycated mAbs above the original mAbs (mAbs before glycation treatment). The heatmap shows that the glycated mAbs induced little to no increase in cytokine secretion or number of IFN-γ secreting cells in both adherent monocytes and PBMC. For the few donors that did show a response, the magnitude of the response was very low (SI<9%, except for the adherent monocytes from two donors which were slightly higher) in all cases (data not shown). This was in contrast to the strong signal induced by the LPS and PHA positive controls, which induced a response in all donors (100%) and a SI of 200–7000+ above background for at least one cytokine per donor. In addition, the response of adherent monocytes to LPS at the early phase was higher than the response of PBMC in terms of percentage of responding donors (Fig 5) and magnitude of the response (data not shown), indicating that adherent monocytes may provide a more sensitive readout than PBMC. Overall, these *in vitro* results suggest that the increased levels of glycation tested do not pose an increased potential risk of immunogenicity. These results are limited, however, by the sensitivity of this assay, which is currently being investigated.

### The IVCIA Assay can be used for Lot-to-lot and Biosimilar Comparability

**Fig 6:**
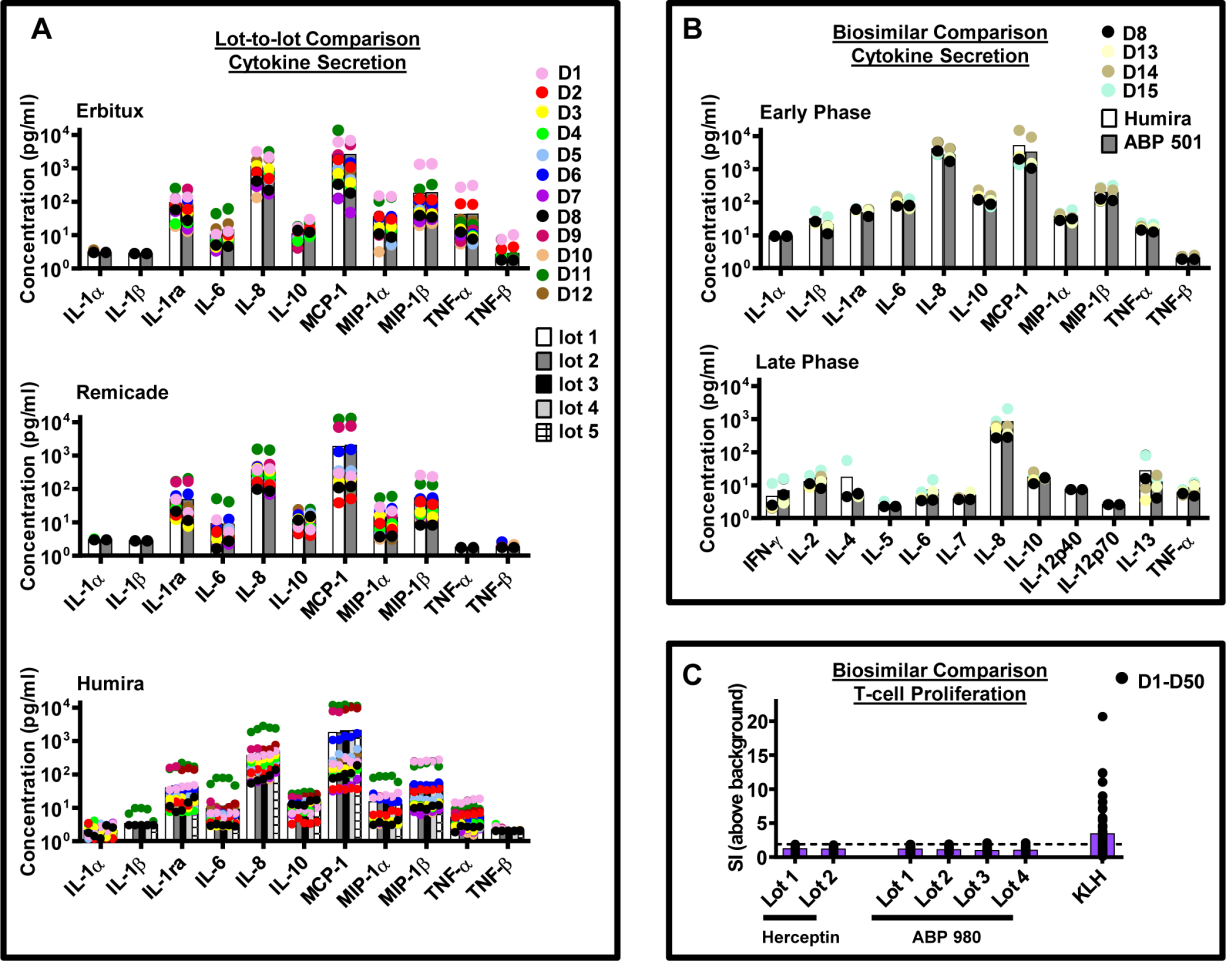
The IVCIA assay can be used to compare mAb lots from both the same manufacturer and from different manufacturers (biosimilars). A) Several different lots of Erbitux (2 lots), Remicade (2 lots) and Humira (5 lots) were tested in the IVCIA assay for a response at the early (20 h) phase (n = 12 donors), at 40 μg/mL. The concentration (pg/mL) of signature cytokines that were secreted 20 hours after stimulation is shown. B) Biosimilars, Humira and ABP 501, from two different manufacturers were compared in the IVCIA assay for the secretion of signature cytokines at the early (20 h) and late (7 day) phases (n = 4 donors), at 100 μg/mL. C) Multiple lots of another set of biosimilars, Herceptin and ABP 980, from two different manufacturers (which are both associated with a low rate of clinical immunogenicity), were assessed in the IVCIA assay at 40 μg/mL for T-cell proliferative responses on Day 7 only. No statistically different responses in the assay were observed between different lots of Herceptin and mAb5-B (p = 0.12). In all assays, the average response across the population tested was similar, although slightly different responses in specific donors could be observed. In all panels, *bars in shades of white and grey* show the average level of cytokine secretion at the early and late phases, and *purple bars* depict the average level of T-cell proliferation, across the population. *Colored and black circles* represent the response of individual donors and show the variability of the population tested.

To test if the IVCIA assay is suitable for comparability assessments, the response to several lots of the same mAb, from both the same manufacturer and different manufacturers (biosimilars), were tested. Fig 6 shows several case studies where the response in PBMC to a variety of different samples, at both the early and late phases, was compared. Fig 6A compares the levels of the secreted signature cytokines from PBMC when exposed to different lots of 3 mAbs (Erbitux, Remicade, and Humira), from the same manufacturer, at the early phase (n = 12 donors). These lots induced an overall similar cytokine secretion profile when averaged across the population at the early phase (Fig 6A, *white and grey bars*), although donor to donor differences could be observed (Fig 6A, *colored circles*). Some donors showed slight differences in response to different lots of the same mAb (compare the response of the same colored dots in Fig 6A), which might be due to differences in attributes since there are no differences in sequence between lots being compared. The consistency of the average cytokine secretion profile across the population demonstrates the reproducibility of the response at the early phase.

The response of the IVCIA assay to biosimilar mAbs was also tested and found to give similar responses across the population. Fig 6B shows that biosimilars Humira and ABP 501 (produced by different manufacturers), induced comparable levels of cytokine secretion (n = 4). Furthermore, no statistical differences between these biosimilars (p≥0.05) were observed for most of the 23 cytokines that were tested at both the early (IL-1β, IL-1ra, IL-6, MIP-1α, MIP-1β, TNF-α, and TNF-β) and late (IL-10, IL-13, IL-2, IL-4, IL-5, IL-6, IL-7, IL-8, and TNF-α) phases. Three cytokines (IL-1α, IL-12p40, and IL-12p70) were at a comparably low concentration in both biosimilar samples, however they could not be statistically analyzed as the cytokine concentration was at the limit of quantitation. Statistical differences (p<0.05) were observed for a few remaining cytokines at the early (IL-8, IL-10, and MCP-1) and late (IFN-γ) phases, however the stimulation index between the two products was well below the cutoff used to determine a positive response in the assay (SI ≥ 1.9–2.0) for all cytokines, indicating that were indeed comparable. Overall, although differences in donor specific responses could be observed (Fig 6B), the differences were similar to those observed for unique lots of the same mAb from one manufacturer (Fig 6A). Another pair of biosimilars from two different manufacturers, Herceptin and ABP 980 (which have low rates of clinical immunogenicity), were tested in a single time point assay (Day 7 only) for T-cell proliferation in a 50 donor population. All lots of Herceptin and ABP 980 that were tested showed a comparably low response in the assay (Fig 6C), and were not found to be statistically different (p = 0.12), which agrees with the low rate of clinical immunogenicity for this molecule.

## Discussion

The responses observed in the IVCIA assay were aligned with the relative clinical responses of the 10 mAbs tested. The assay may help rank order and select therapeutic mAb candidates that would be expected to have the least potential to be immune activating in the clinic based on their ability to drive a T-cell functional response *in vitro*. For example, mAb therapeutics such as Xolair and Herceptin, with low T-cell responses (≤10% of donors responded), would be ranked low on the scale for potential immunogenicity risk. Similarly, Remicade and Humira, that showed higher T-cell responses (≥10% of donors responded), will be ranked toward the right side of the scale with a higher risk for potential immunogenicity (>10% of donors responded) (Table 1). This threshold between biotherapeutics with lower and higher risk of immunogenicity is in agreement with other published results, where molecules with a low rate of clinical immunogenicity induced less than 10% of donors to respond in the assay [5,6,8]. The primary readout of the IVCIA assay is T-cell proliferation; however this includes the responses of both T-effector and T-regulatory cells. As T-regulatory cells do not secrete IL-2 [29], two orthogonal readouts, number of IL-2 secreting cells and concentration of IL-2 secreted, were also used. This helped to confirm that the T-cell profiles observed were associated with an inflammatory output that could eventually drive an antibody response. Therefore, for mAb rank ordering and therapeutic selection, the use of the IVCIA assay with at least two orthogonal readouts (T-cell proliferation and either number of IL-2 secreting cells or concentration of IL-2 secreted) is suggested.

There are multiple operational benefits to these types of cell-based assays including the ability to: screen the same donor with a large number of samples, test a variety of different sample types (i.e. highly stressed) that may not be safe to test in the clinic, achieve a production level that is not possible with *in vivo* experiments, retrospectively assess manufacturing changes, and potentially operationalize in a manufacturing setting. Additional advantages include that these assays can: incorporate the HLA diversity of the human population, be evaluated in human donors with a known medical history, simulate potential immune cell activation, and contain a relevant mixture of immune cells without the confounding influence of other components of tissues and blood [22,23].

Although the IVCIA assay is useful for relative risk ranking and candidate selection, the utility of this assay is limited by several factors. It is notable that these assays lack many of the components that may contribute to an immune response *in vivo*, including: the route of administration and disposition of the biologic, processing of the biologic by the relevant professional antigen presenting cells, buffering of the response by the presence of other relevant cell types in physiologically relevant ratios, and the interaction with surrounding tissues and lymph nodes [22,23]. Even though the assay was effective at identifying the likelihood of mAbs to be potentially immunogenic in the clinic, the assay cannot be used for predicting the rate of immunogenicity in the clinic. Furthermore, actual immunogenic potential may only be measured via a multi-dose clinical assessment. It is important to note that the assay does not account for the disease state and underlying immune status as well as standard care of treatment of patients that can modulate such responses. Additionally, the anti-drug antibody assays used to measure clinical immunogenicity are not harmonized across industry and face technical limitations of low sensitivity, specificity, and interference due to circulating mAbs or other soluble factors [23]. Therefore, using the observed immunogenicity incidence numbers from the label of the marketed products as well as from published clinical studies should be interpreted with caution. Another exception to this analysis is the observed or clinical immunogenicity rates of biotherapeutics with an immunomodulatory target (i.e. Campath, Rituxan, or therapeutics with cytokine/chemokine targets [30]). For these types of molecules, the level of T-cell response detected *in vitro* may not be consistent with immunogenicity observed in the clinic [31].

**Fig 7:**
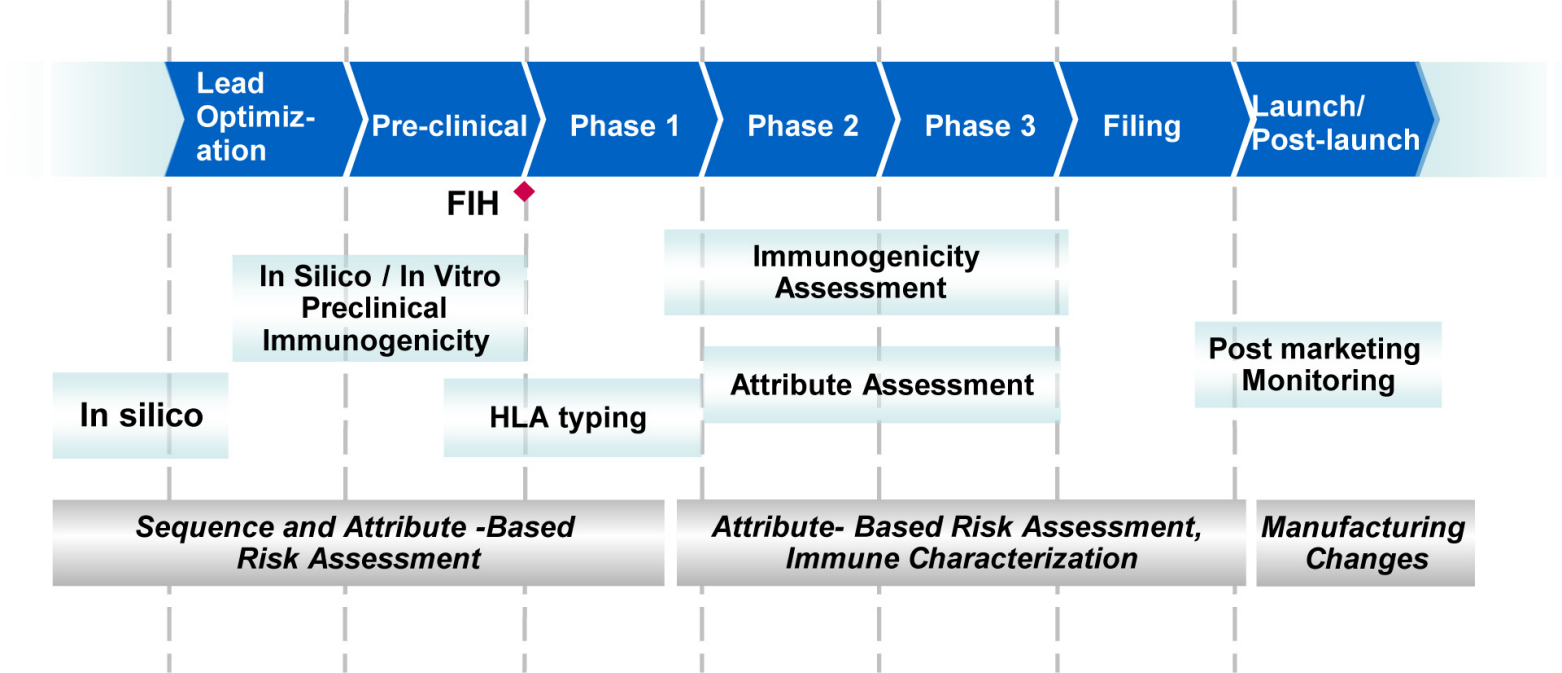
Stages of biotherapeutic development where the IVCIA assay can be used for risk assessment related to attributes. *In silico* or algorithm based assessments rank order and identify lead candidates based on the least sequence based risk. *In vitro* assessments identify non-sequence attributes and any immune mediated target based risk at preclinical stage prior to first in human (FIH). Pharmacogenomic assessments for HLA can be introduced in long-term clinical studies (Phase 1b/2) to evaluate associations of HLA with clinical immunogenicity. Immunogenicity assessments measured in serum as ADA span the breadth of clinical trials and disease indications (FIH to Launch/post launch). *In vitro* assays also provide attribute related risk assessment of manufactured lots, lot-to-lot comparability, and risk post packaging due to attributes related to storage, shipping, handling, and device-related leachates.

The application of the IVCIA assay to the development of biopharmaceuticals can range from candidate selection at the early development phase to the late stage evaluation of specific attributes that may impact the risk of immunogenicity. Fig 7 shows a proposed schematic for immunogenicity risk assessment of biotherapeutics during the drug product development lifecycle. During candidate selection, there are often multiple variants of a biotherapeutic. For a therapeutic protein with an endogenous counterpart, a comparison between the native protein and the biotherapeutic itself is performed to make sure that the biotherapeutic is as close to the self-protein as possible. Here, a comparison of FP1 to FP1 (no Fc) in the IVCIA assay showed a minor decrease in the T-cell response when the FP1 molecule was fused to an antibody Fc domain. However the assay was the most meaningful in distinguishing the immunogenic potential of the mutations associated with the FP1 molecule itself. An *in silico* evaluation could not identify and rank order the variants as they are all predicted to have a similar risk of immunogenicity. A rank ordering of the FP1 candidates from highest to lowest risk (with FP1 mutant 1 > FP1 mutant 2 > FP1 mutant 3) was achieved by the IVCIA assay, confirming that minor mutations in the sequence, that were not differentiated by *in silico* analysis alone, could be detected by the T-cell functional assay. Although the different FP1 mutants did show differences in a few additional attributes (including the aggregation profile) which might impact the response in the IVCIA assay, the assay is capable of detecting both differences in sequence and differences in attributes that result from changes in the sequence. The sensitivity of T-cells *in vitro* to minor changes in sequence in viral antigens has been shown by others [32]. However, the ability of the assay to detect differences in sequence in other protein molecules may depend on the exact location and nature of the variation in sequence.

During later stage development, the potential risk of immunogenicity due to attributes that can vary due to the manufacturing process (such as protein aggregation and chemical modification) [8,33–37] becomes more evident. Protein aggregates in the sub-visible size range is one attribute that has been suspected of posing an increased risk of immunogenicity [4,22,38–41]. The original and highly aggregated forms of 10 diverse mAbs were tested in the IVCIA assay for changes in immune functional response at the early and late phases. The question posed was whether the aggregated forms of these mAbs would all be able to activate the immune cells in the assay. Although each mAb tested had an individual sequence-associated or target-related immunogenic potential, when aggregated, all but one mAb showed a similar trend of being higher in their functional response than the original mAb. This is consistent with published results that show that high numbers of aggregates in the 2–10 μm size range (far above that found in marketed products) have the potential to activate immune cells *in vitro* and induce weak and transient responses *in vivo* [8–10,16,21,22,42]. This enhanced response due to aggregation was detected by measuring a panel of signature cytokines and could be observed as soon as the early phase, indicating that a single readout at the early phase may be sufficient for detecting the potential risk of immunogenicity due to attributes. Therefore, a risk of immunogenicity scoring system could be devised by calculating the percentage of donors that responded by one or more readouts in the assay.

Herceptin, Xolair, Erbitux, and Avastin samples containing high levels of aggregates induced the highest response in the assay, although the mAbs before aggregation were not strong elicitors of a T-cell functional response. Furthermore, mAbs that caused a higher T-cell functional response, such as Remicade, mAb3 and Humira, were only slightly more immunogenic when stress treated. The lower response could be attributed to the more immunogenic nature of these molecules as observed by their higher T-cell functional responses before stress treatment (>10% donors responded). The response to aggregates could be detected as early as 20 hours post-stimulation as shown by increased levels of monocyte-specific cytokines (S2 Fig), indicating the involvement of monocytes, macrophages and immature dendritic cells. The same aggregates were also able to elicit a T-cell functional response at a later stage (7–8 days). In most cases, donors that responded significantly at the late stage (SI≥5) had also induced a response at the early phase. Our previously published results in the transgenic Xeno-het mouse model showed that the response to high numbers of aggregates is transient and weak and not associated with long-term memory [42]. Hence, the initial innate phase activation could lead to engagement of antigen presenters that could potentially drive a weak T-cell response at the late phase.

Overall, this indicates that the relative response to aggregates in the assay does not correlate with the rate of clinical immunogenicity of the unstressed molecule. Aggregates may possess unique structural features or 3-dimensional epitopes that can lead to B-cell or T-cell activation (perhaps through enhanced uptake and subsequent activation of antigen presenting cells) in a manner that is not dependent on the T-cell epitope content of the linear amino acid sequence. In addition, two of the aggregated samples that induced the highest response (Herceptin and Avastin) did not have the highest number of particles present (Avastin was one of the lowest) suggesting that additional aggregate attributes (besides aggregate number) are contributing to the immune response observed. The assay also demonstrated the target mediated effect of Campath on T-cells. Campath, both before and after aggregation, showed no response in the assay, suggesting that the monomeric (or monomer fraction of the stirred sample that was not aggregated upon stress treatment) and/or aggregated Campath retained its ability to bind to the target in the assay. It is important to note that all of these aggregated samples are heavily aggregated and have particle numbers that are orders of magnitude above what would be in a marketed product. Future experiments that evaluate the threshold of aggregate activation in the IVCIA assay will be key to establishing the sensitivity of the assay. Further correlation between the threshold of activation in the IVCIA assay to the threshold of response in humans will be necessary for setting biologically relevant specifications during product manufacturing.

Glycation is the non-enzymatic addition of sugars to proteins and occurs on lysine side chains and the N-terminal amine. Glucose is traditionally used as a carbon source during mammalian cell culture expression, although other sugars could be used. Glycation levels of antibody biotherapeutics are product quality attributes that can vary during the expression and manufacturing of the drug product, or after administration to patients *in vivo* [24,43]. Although there is a theoretical risk of activation of antigen presenters, such as monocytes or dendritic cells through mannose receptors [44–46], the immunological impact of protein biotherapeutics modified by glycation with different sugars is unknown. The IVCIA assay was used to explore the impact of mAbs with elevated levels of glycation by different sugars (galactose, glucose, and mannose), to approximately 40% above the original mAbs, on the potential risk of immunogenicity. At both the early and late phases, glycation of mAbs showed a very low or negligible response. Some donors were associated with a low level response that could be attributed to the inherent biological state of the donor as compared to other healthy subjects. Overall, antibody glycation from galactose, glucose, and mannose at the levels tested does not appear to trigger activation of a potential immune reaction, as judged by cytokine release assays of certain immune cells *in vitro*.

The suitability of the IVCIA assay for comparing various lots of the same mAb, from both the same manufacturer and from different manufacturers (biosimilars), was demonstrated. The different lots that were compared had the same sequence but contained varying levels of certain product quality attributes. For example, Humira lots varied in several characteristics including: some N-linked glycan species (high mannose, non-fucosylated complex glycans, degree of galactosylation of complex glycans, and degree of sialylation of complex glycans) and number of sub-visible particles in the 2–10 μm and greater than 10 μm size ranges (data not shown). It is important to note that Humira is administered in a pre-filled syringe so particle numbers showed high variability between lots and are mostly silicone oil droplets (>98%). As another example, Erbitux lots also showed variability in some N-linked glycan species (high mannose, non-fucosylated complex glycans, and degree of sialylation of complex glycans) and % high molecular weight by size exclusion chromatography (data not shown). The IVCIA assay showed a similar average profile across the population to different lots of the same mAb, although individual donor to donor differences could be observed. The IVCIA assay may therefore be an important comparability technique for showing both high-level similarity and detecting minor attribute-related differences.

In conclusion, the IVCIA assay is an informative cell-based system for relative immunogenicity risk assessment, and has multiple applications from early development through the lifespan of the drug product.

## Supporting Information

S1 Fig The distribution of donor allotypes used in the IVCIA assay was similar to that expressed in the world population. The HLA-DR allotypes of one representative set of 50 donors that was used in the assay is shown. Donors were selected to best represent the number and frequency of HLA-DR allotypes expressed in the world population.
(PDF)

S2 Fig Aggregated biotherapeutic mAbs enhance cytokine secretion above the original mAb in the IVCIA assay at both the early and late phases. PBMC from 50 human donors were challenged with the original and aggregated forms of several biotherapeutic mAbs. Multiplex cytokine analysis was performed to evaluate the level of secretion of signature cytokines after A) the early phase (20 h) and B) the late phase (7 days) post challenge. The average SI of positive donors at the early phase (SI ≥ 2.0, yellow bars) or late phase (SI ≥ 1.9, green bars) and percentage of donors that responded (% donors, grey bars) to the aggregated mAb above the original mAb is shown. Representative cytokines that displayed the strongest responses are shown. Asterisks (*) highlight statistically significant differences (p<0.05). Black circles depict responding individuals and highlight the distribution of responses across the population tested.
(PDF)

S3 Fig Biotherapeutics before and after aggregation by stirring stress stimulate the secretion of IL-10. Donors that were positive for T-cell proliferation in the IVCIA assay over the entire study (5–8 days) in response to A) the original mAbs or B) aggregated mAbs at the late phase were evaluated by multiplex cytokine analysis for the secretion of IL-10 on Day 7 (n = 50 donors). Not all donors were tested for IL-10 for some samples (grey circles). The percentage of donors that responded positively in the proliferation assay (purple bars) and the percentage of donors that responded positively for both proliferation and the secretion of IL-10 (green bars) are shown. A response was considered positive if the SI ≥ 2.0 (p<0.05) for proliferation or the SI ≥ 1.9 for IL-10 concentration (above the background response). The asterisk indicates that borderline T-cell responses were included (SI ≥ 1.9) in some cases. The scale bars at the top of each graph show the relative rate of clinical immunogenicity taken from the product label (see Table 1). All rates are associated with diverse disease indications and assay testing platforms with variable sensitivity.
(PDF)
