# Supplementary figures and images for: Use of In Vitro Assays to Assess Immunogenicity Risk of Antibody-Based Biotherapeutics
Source: PLoS One. 2016 Aug 5;11(8):e0159328. doi: 10.1371/journal.pone.0159328 (PMC4975389; doi:10.1371/journal.pone.0159328)

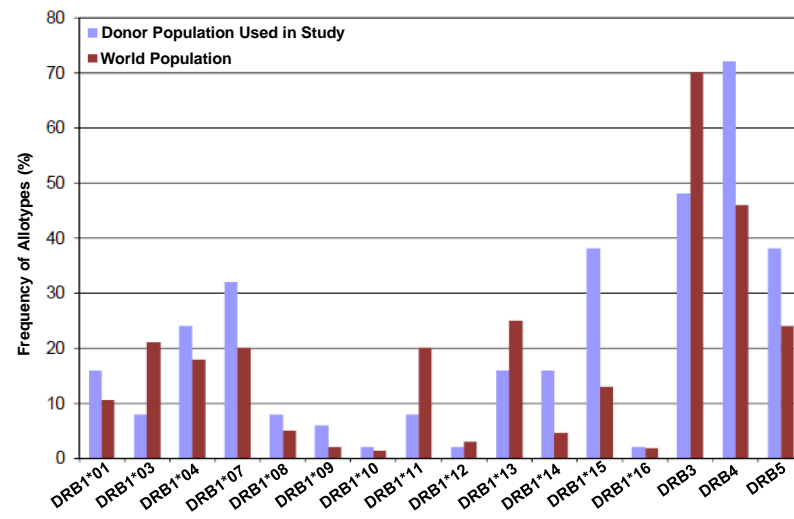

**S1 Fig**

Supplement: S1 Fig — The HLA-DR allotypes of one representative set of 50 donors that was used in the assay is shown. Donors were selected to best represent the number and frequency of HLA-DR allotypes expressed in the world population. (PDF) [file pone.0159328.s001.pdf]

### A Early (20 hour) Response

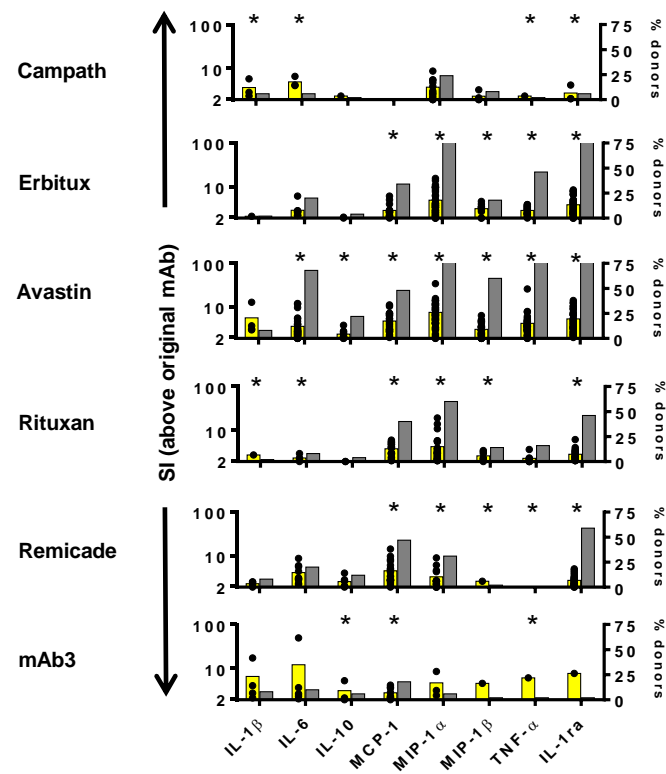

### B Late-stage (7 day) Response

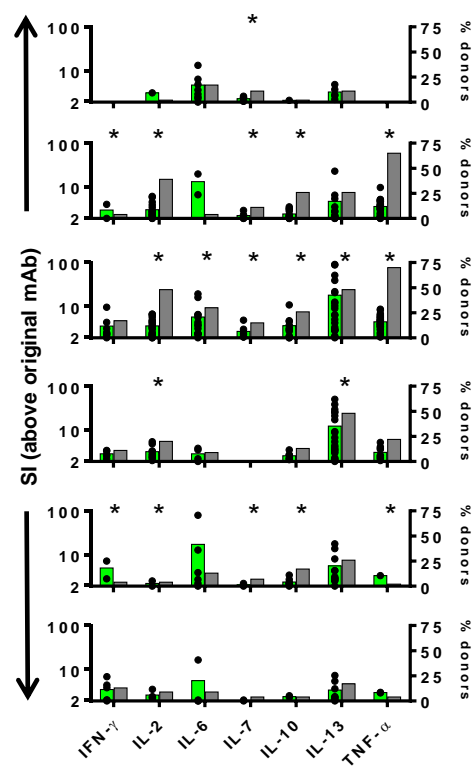

S2 Fig

Supplement: S2 Fig — PBMC from 50 human donors were challenged with the original and aggregated forms of several biotherapeutic mAbs. Multiplex cytokine analysis was performed to evaluate the level of secretion of signature cytokines after A) the early phase (20 h) and B) the late phase (7 days) post challenge. The average SI of positive donors at the early phase (SI ≥ 2.0, yellow bars) or late phase (SI ≥ 1.9, green bars) and percentage of donors that responded (% donors, grey bars) to the aggregated mAb above the original mAb is shown. Representative cytokines that displayed the strongest responses are shown. Asterisks (*) highlight statistically significant differences (p<0.05). Black circles depict responding individuals and highlight the distribution of responses across the population tested. (PDF) [file pone.0159328.s002.pdf]

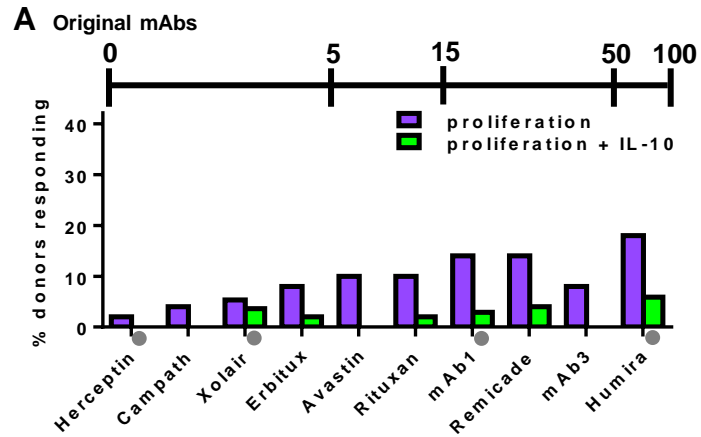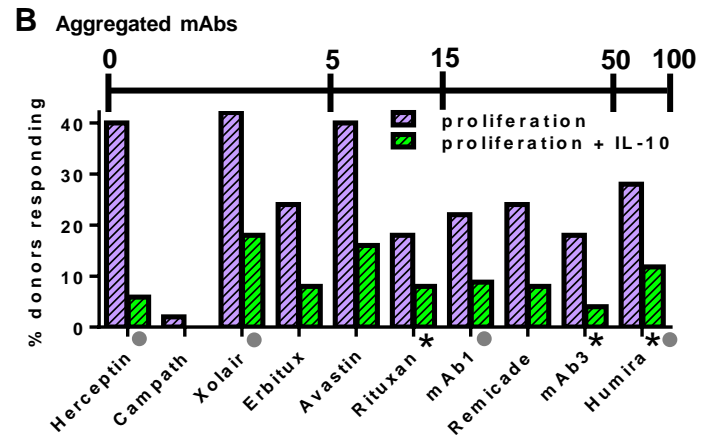

**S3 Fig**

Supplement: S3 Fig — Donors that were positive for T-cell proliferation in the IVCIA assay over the entire study (5–8 days) in response to A) the original mAbs or B) aggregated mAbs at the late phase were evaluated by multiplex cytokine analysis for the secretion of IL-10 on Day 7 (n = 50 donors). Not all donors were tested for IL-10 for some samples (grey circles). The percentage of donors that responded positively in the proliferation assay (purple bars) and the percentage of donors that responded positively for both proliferation and the secretion of IL-10 (green bars) are shown. A response was considered positive if the SI ≥ 2.0 (p<0.05) for proliferation or the SI ≥ 1.9 for IL-10 concentration (above the background response). The asterisk indicates that borderline T-cell responses were included (SI ≥ 1.9) in some cases. The scale bars at the top of each graph show the relative rate of clinical immunogenicity taken from the product label (see Table 1). All rates are associated with diverse disease indications and assay testing platforms with variable sensitivity. (PDF) [file pone.0159328.s003.pdf]
